# Development of Indicators for Patient Care and Monitoring Standards for Secondary Health Care Services of Mumbai

**DOI:** 10.1371/journal.pone.0119813

**Published:** 2015-03-17

**Authors:** Seema S. Malik, Roshni Cynthia D’Souza, Pramod Mukund Pashte, Smita Manohar Satoskar, Remilda Joyce D’Souza

**Affiliations:** 1 Secondary Health Care Services, Municipal Corporation of Greater Mumbai, Mumbai, Maharashtra, India; 2 Information Technology Consultant, Mumbai, Maharashtra, India; Texas Tech University Health Science Centers, UNITED STATES

## Abstract

**Background:**

The Qualitative aspect of health care delivery is one of the major factors in reducing morbidity and mortality in a health care setup. The expanding suburban secondary health care delivery facilities of the Municipal Corporation of Greater Mumbai are an important part of the healthcare backbone of Mumbai and therefore the quality of care delivered here needed standardization.

**Material and Methods:**

The project was completed over a period of one year from Jan to Dec, 2013 and implemented in three phases. The framework with components and sub-components were developed and formats for data collection were standardized. The benchmarks were based on past performance in the same hospital and probability was used for development of normal range. An Excel spreadsheet was developed to facilitate data analysis.

**Results:**

The indicators comprise of 3 components - Statutory Requirements, Patient care & Cure and Administrative efficiency. The measurements made, pointed to the broad areas needing attention.

**Conclusion:**

The Indicators for patient care and monitoring standards can be used as a self assessment tool for health care setups for standardization and improvement of delivery of health care services.

## Introduction

**Fig 1:**
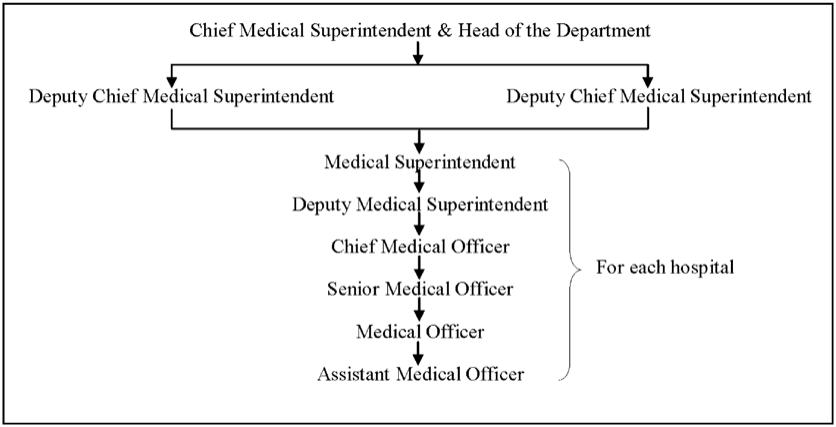
Hierarchy of administration at Peripheral Hospitals (Secondary Health Care). The administrative hierarchy is outlined which gives an understanding of the workforce at Peripheral Hospitals in Mumbai.

The urban governance of the metropolitan city of Mumbai is done by the Municipal Corporation of Greater Mumbai (MCGM) established in 1882, India’s first Municipal Corporation. The civic body has been foremost in providing all public services including health services. The planning and development of health infrastructure in the metropolis has been an ongoing process started over a century back. The MCGM health infrastructure includes three major teaching medical colleges and hospitals and one dental college and hospital forming the tertiary group of health delivery services located within the city. The secondary health care services are provided by 18 peripheral multispecialty hospitals located in suburbs and extended suburbs. Hierarchy of administrative control of the 18 peripheral hospitals providing secondary care is shows in Fig. 1. Besides this, primary health care is provided by health posts, dispensaries, Maternity Homes, Post partum centres. In addition there are five specialized hospitals for Tuberculosis, Leprosy, ENT, Eye Care and Infectious diseases.

The health infrastructure not only caters to a population of 12.4 million (as per 2011 Census) over an area of 480.24 sq.km but also to the population coming from all over the state and country for availing of the broad health care services provided at these institutions. This has always lead to increased public expectations from the existing resources which has driven expansion policies for hospitals and also the setting up of new hospitals in the area of secondary health care. The bed strength and performance for the Secondary Health Care services in Mumbai are shown in Table 1 and 2 respectively. Political commitments and advocacy have partly ensured the quantitative aspect of meeting the enormous health infrastructure needs. Meanwhile, the Administrators in addition had to make concerted efforts to meet the qualitative aspects of service delivery leading to the inception of Indicators for Patient Care and Monitoring Standards.

**Table 1:** Peripheral Hospitals (Secondary Health Care)—Total Bed strength (4584).

	Western Suburbs	Beds	Eastern Suburbs	Beds
**Major Hospitals**	Khusheedji Beheramji Bhabha Hospital, Bandra	436	Seth V C Gandhi & M A Vora Rajawadi Hospital, Ghatkopar	586
	Dr. R. N. Cooper Hospital, Juhu *(Redeveloped hospital commissioned on 30* ^*th*^ *Nov*., *2013)*	636	Khan Bhadur Bhabha Hospital, Kurla	306
	Hinduhridaysamrat Balasaheb Thackeray Trauma Care Hospital, Jogeshwari *(Commissioned on 21* ^*st*^ *Oct*., *2013)*	304		
	Bharatratna Dr. Babasaheb Ambedkar Hospital, Kandivali *(Commissioned on 02* ^*nd*^ *Sept*., *2013)*	324		
	Shri Harilal Bhagwati Hospital, Borivali	373		
**Mid-sized hospitals**	V. N. Desai Hospital, Santacruz	254	Pandit Madan Mohan Malviya Hospital, Govandi	210
			Smt. Mansadevi Tulsidas Agarwal Hospital, Mulund	225
**Small-sized Hospitals**	Siddharth Hospital, Goregaon	172	K. M. J. Phule Hospital, Vikhroli	140
	S. K. Patil Hospital, Malad	50	S. V. D. Savarkar Hospital, Mulund	105
	M. W. Desai Hospital, Malad	180	St. Muktabai Hospital, Ghatkopar	104
	Municipal General Hospital, Kasturba Cross Road, Borivali	105	MAA Hospital, Chembur	74
	**Total Beds**	**2834**	**Total Beds**	**1750**

**Table 2:** Performance of the Peripheral Hospitals (Secondary Health Care) in terms of cases attended.

	Annual(Jan-Dec, 2013)	Average per day
O.P.D. Attendance	46,30,566	15,435
Number of Casualties Attended	7,98,295	2,187
Number of Admissions	2,18,285	598
Number of Deliveries	28,800	79
Number of Surgeries	32,289	88

## Material and Methods

**Fig 2:**
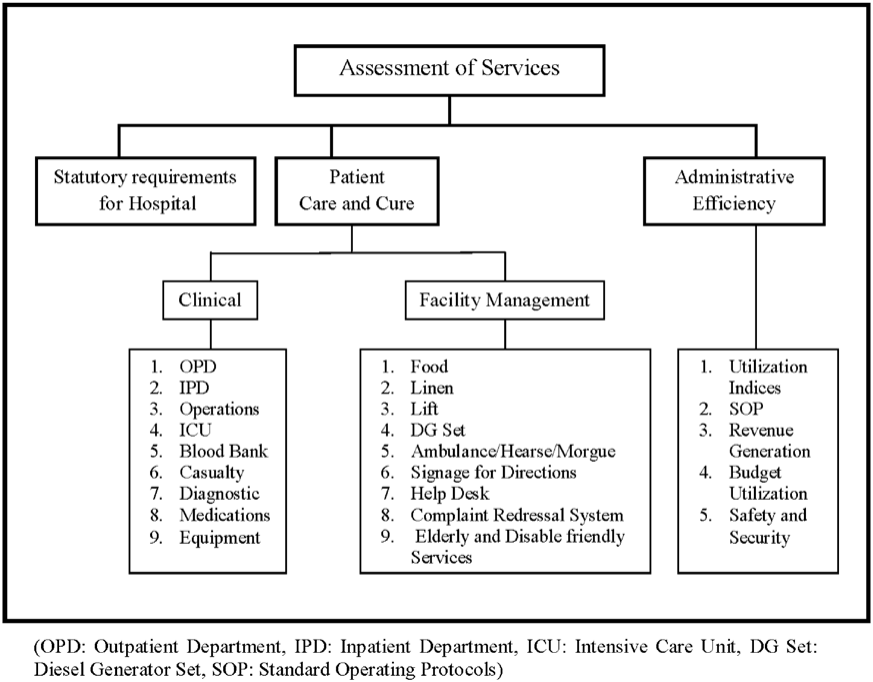
Structural Framework for Assessment of Hospital Performance. The structural framework is the basis on which the indicators for patient care and monitoring standards are based.

The study was conceptualized in Jan., 2013 and was developed over a period of one year by Dec., 2013. The study was approved by the Hospital Ethics Committee. Only number of events recorded in the hospitals was studied and the data was analyzed anonymously, therefore no consent was taken. Also, the research only involves the study of the process of development of standards and no data about human subjects is reported in any section. During the inception, only 16 peripheral hospitals existed. Two new hospitals and one redeveloped hospital were commissioned between Sept., 2013 and Dec., 2013 (Table 1). Extensive review of literature of various international and national attempts at developing standard parameters for hospital performance brought forth the unique nature of assessing the unequal sized peripheral hospitals run by a public sector organization. Therefore, key informant interviews which included Medical Superintendents (administrative heads of each hospital), MCGM Engineers, Nursing in-charges, Medical Records officers and clerical staff were conducted. A frame work of indicators was developed. This was then subject to several brain storming sessions wherein stakeholders as well as external agencies were invited. The framework was revised and only relevant parameters were included and bench-marking was done for the parameters. Simultaneously, a base line survey was conducted for studying existing system of data management in the peripheral hospitals. Independent external advisory agencies were asked to comment on the framework developed (Fig. 2). The findings were then presented to the Municipal Commissioner of Mumbai. This was followed by field testing in the form of a pilot study involving only four major hospitals, namely Rajawadi Hospital, Shri Harilal Bhagwati Hospital, Dr. R.N. Cooper Hospital and Khusheedji Beheramji Bhabha Hospital. The project was implemented in phases. In the first phase the pilot study was done, in the second phase eight hospitals were included and in the third phase all the hospitals were included. In the pilot study, data was filled by the various departments of each hospital in an excel sheet and this was analyzed by the principal investigators at the level of the office of the Chief Medical Superintendent and the report was disseminated to the hospitals and a consolidated copy was sent to Additional Municipal Commissioner holding health portfolio and her approval was obtained. The pilot study brought forth the need for structured data maintenance at user-level which was addressed by one day workshops in four batches which resulted in development of formats for data by users themselves, thus bringing about involvement of representatives of staff of these hospitals.

**Fig 3:**
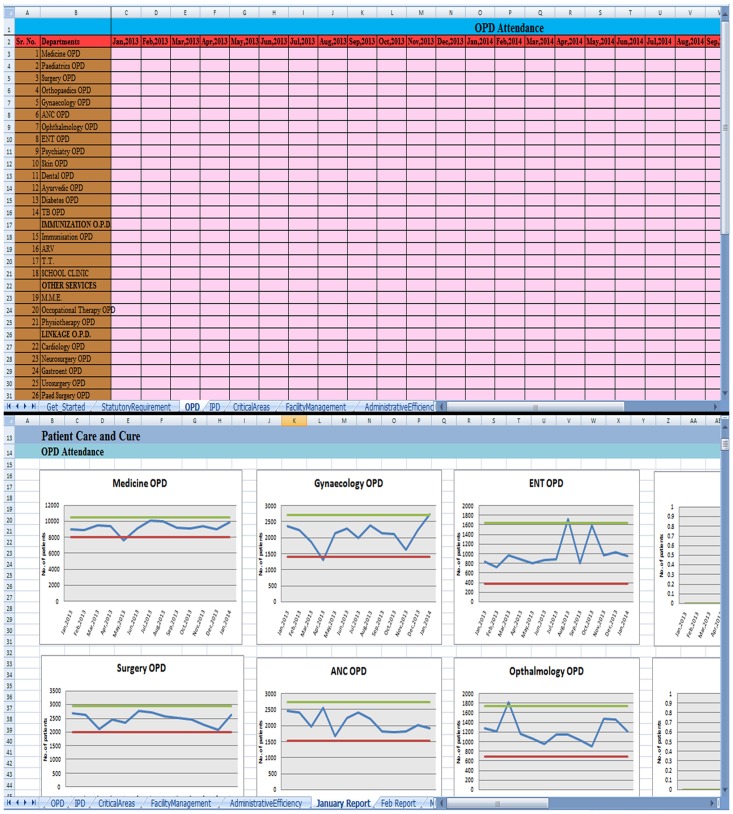
Excel spreadsheet: User-friendly Tool with Graphical representation of the parameters. The Top half depicts the data entry sheet. The Lower half is the sheet displaying the graphs generated on entering data in the data entry sheet. The graphs have three trend lines; upper green and lower red lines represent two standard deviations above and below the mean of the previous one year’s observed statistics respectively, the middle blue line represents the current monthly findings of that particular parameter.

In the pilot study, it was noticed that collection, compilation, analysis of data and report writing was a labor intensive activity and it would be highly difficult to sustain the project on a long term basis and projection of the project to all 18 peripheral hospitals would develop an enormous bank of data, the analysis of which would, not only be tedious but also timely report generation would be affected defeating the main purpose of the project. Therefore, the in-house team developed a simple user-friendly excel spread sheet which only required the users to fill in data and analyzed graphs would be generated (Fig. 3). The staff of peripheral hospitals were trained in filling the excel spreadsheet and interpreting the graphs. The project was then extended to all peripheral hospitals.

## Results

The framework (Fig. 2) developed for the indicators comprises of three components-Statutory Requirements for the hospital, Patient care and cure and Administrative efficiency.

Component 1: Statutory Requirements includes licenses, and certifications which are essential to permit hospitals to run. All indicators under this component are to be present, absence or non-renewal of any one amounts to a high priority need for intervention by the administration. The excel spreadsheet was designed to signal a caution three months before the date of expiry of license.

Component 2: Patient Care & Cure includes two sub-components—Clinical and Facility Management. The benchmarking for the indicators in the first sub-component was based on the previous years’ performance at the same hospital. The normal range was taken as mean ± 2 standard deviations of the previous one year record and for some indicators a comparison was made with the median of the data of the previous one year. For the sub-component facility management, the benchmarking was done so as to minimize patient inconvenience.

Component 3: Administrative efficiency included indicators—Operation Theatre utilization, Budget utilization, Revenue generation, availability of core and critical medicines, staff vacancy position, pathological investigations and adherence to the Standard Operating Protocols, that were developed to guide and allow systematic manner of discharge of duties.

Based on these components, the indicators allowed administrators to pick up early, the downward trend or below-normal level (p = 0.05) of performance of services. The indicator then depicted the area of concern which needed attention and further investigation to locate the problem. The problem then was resolved by the administrator.

## Discussion

The indicators developed worldwide have mainly focused on performance relative to other hospitals or against fixed standards. The principal methods of measuring hospital performance have been regulatory inspection, public satisfaction surveys, third party assessment comparison of statistical indicators and internal assessments [1]. However, application of these methods for assessing secondary health care services with varying bed capacities and unequal facilities is of uncertain benefit. To ascertain the optimum functioning of the hospitals with the available facilities is being addressed by the parameters developed in the present project based on the hospital’s past performance. This principle is also the basis for accreditation by National Accreditation Board for Hospitals (NABH). A few studies have even used composite index as a scoring method [2]. The composite index method also may be used, however comparability of the hospital that will be ranked based on the index is essential. This can be done by grouping similar capacity hospitals forming peer groups. The burden of data collection and resources required has been found as a major deterrent to implement most performance parameters and scoring systems. The PATH project stated that lack of personnel, resources, expertise and time for participating hospitals was an issue [3]. Therefore, in the current project the indicators were chosen in a manner in which, their collection would not burden the existing system. The indicators developed though do not directly reflect processes and outcomes in terms of quality of care but they reflect the broad areas which would be sensitive to changes in quality of care. The excel spreadsheet is used as a tool to decentralize the interpretation and report writing at the hospital level so that only those findings that need the intervention or knowledge of the higher authorities can be reported to them. This involved only minimal expenditure, thereby being mainly applicable to low resource setups.

The limitations of the indicators developed included that certain primary data like wait times, economic analysis, infection control that required additional resource expenditure was not collected. Bench-marking done is based on the previous one year performance of the same hospital. No comparison is being made in-between hospitals and therefore standardization and comparability of hospitals cannot be done.

Quality of Services rendered in a hospital is an essential aspect which affects patient outcome. In this study, a self-assessment based benchmarking tool was used to assess the hospital performance. The indicators for patient care and monitoring standards, is a useful, low-cost, user friendly tool, which may be used for getting an overview of the performance of the hospital and thereby can aid in decision making. Although further development is needed to include more detailed parameters which may be employed towards getting to a standard optimized universal model in Quality Health Care Service Delivery.

## Supporting Information

S1 File Excel spreadsheet for Data Entry and Report Generation with Graphical Depiction of the Parameters. (XLS)

S2 File Instructions to fill Excel spreadsheet. (DOC)
