# Supplementary material for: Development of Indicators for Patient Care and Monitoring Standards for Secondary Health Care Services of Mumbai
Source: PLoS One. 2015 Mar 17;10(3):e0119813. doi: 10.1371/journal.pone.0119813 (PMC4364454; doi:10.1371/journal.pone.0119813)
Supplement: S2 File — (DOC) [file pone.0119813.s002.doc]

**INSTRUCTIONS TO FILL EXCEL SPREADSHEET**

**Sheets to be filled:**

The following sheets in Excel spreadsheet are to be filled

1. Get started
2. Statutory Requirement
3. OPD (Outpatient Department)
4. IPD (Inpatient Department)
5. Critical Areas
6. Facility Management
7. Administrative efficiency

The sheets are to filled as follows

1. Only pink cells are to be filled
2. Data is to be filled for the previous one year starting from January till the present month.
3. Every month, data of that month is to be entered in the columns for that month in all the first seven sheets

**Formulas:**

A few variables need to be entered after calculation. The formulas for these are as follows:


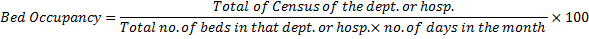


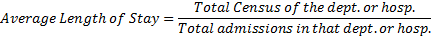


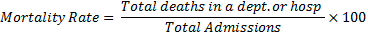


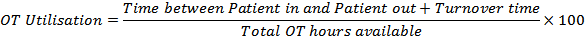


(Abbreviations: **dept.**- department, **hosp.**- hospital, **no.**- number, **OT**- Operation Theatre)

**Report Generation:**

The report of the month will be generated with graphs on the report sheet (eg. for the month of September, sheet of “September Report” is to be seen.) The report sheet is to be interpreted by the administrator and any deviation is to be noted. The cause of the deviation may then be explored.

**Benchmarking:**

- - **Statutory Requirements:** The requirement must be present
  - The Normal Range of a parameter is taken as Mean ±2(Standard Deviations) of previous one year. The graph represents this as two lines, namely the upper limit represented by a green line and the lower limit by a red line. The current level of the parameter is shown by the middle blue/black line. Any dip or rise of this line outside the upper or lower limit lines is taken as a deviation, eg. In the figure below the number of patients attending the Skin Outpatient Department has gone below the acceptable range in the month of Dec.,2013.


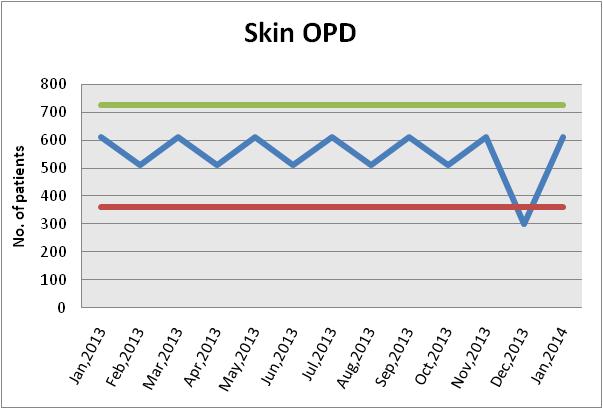


- For certain variables, the median is taken as reference level as the number of observations are few or very variable. In the example below, the central pink line is the median level and the blue line represents the observations seen.


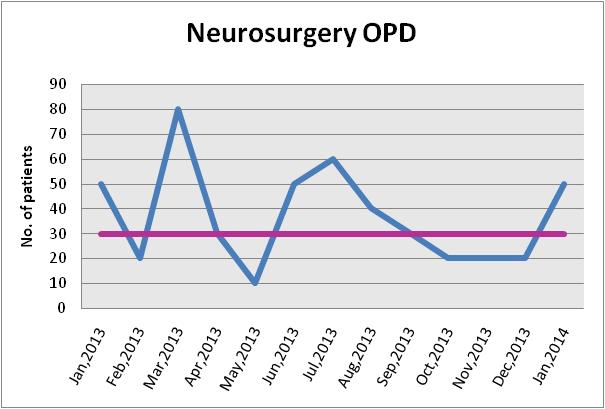


- For the other variables the acceptable range may be fixed based on local settings and later the acceptability standards may be raised to ideal levels.
